# Seasonal Variation in Vitamin D_3_ Levels Is Paralleled by Changes in the Peripheral Blood Human T Cell Compartment

**DOI:** 10.1371/journal.pone.0029250

**Published:** 2012-01-03

**Authors:** Ai-Leng Khoo, Hans J. P. M. Koenen, Louis Y. A. Chai, Fred C. G. J. Sweep, Mihai G. Netea, André J. A. M. van der Ven, Irma Joosten

**Affiliations:** 1 Department of Laboratory Medicine, Laboratory of Medical Immunology, Radboud University Nijmegen Medical Center, Nijmegen, The Netherlands; 2 Department of Medicine, Radboud University Nijmegen Medical Center, Nijmegen, The Netherlands; 3 Nijmegen Institute for Infection, Inflammation and Immunity, Nijmegen, The Netherlands; 4 Department of Medicine, National University Health System, Singapore, Singapore; Blood Systems Research Institute, United States of America

## Abstract

It is well-recognized that vitamin D_3_ has immune-modulatory properties and that the variation in ultraviolet (UV) exposure affects vitamin D_3_ status. Here, we investigated if and to what extent seasonality of vitamin D_3_ levels are associated with changes in T cell numbers and phenotypes. Every three months during the course of the entire year, human PBMC and whole blood from 15 healthy subjects were sampled and analyzed using flow cytometry. We observed that elevated serum 25(OH)D_3_ and 1,25(OH)_2_D_3_ levels in summer were associated with a higher number of peripheral CD4^+^ and CD8^+^ T cells. In addition, an increase in naïve CD4^+^CD45RA^+^ T cells with a reciprocal drop in memory CD4^+^CD45RO^+^ T cells was observed. The increase in CD4^+^CD45RA^+^ T cell count was a result of heightened proliferative capacity rather than recent thymic emigration of T cells. The percentage of Treg dropped in summer, but not the absolute Treg numbers. Notably, in the Treg population, the levels of forkhead box protein 3 (Foxp3) expression were increased in summer. Skin, gut and lymphoid tissue homing potential was increased during summer as well, exemplified by increased CCR4, CCR6, CLA, CCR9 and CCR7 levels. Also, in summer, CD4^+^ and CD8^+^ T cells revealed a reduced capacity to produce pro-inflammatory cytokines. In conclusion, seasonal variation in vitamin D_3_ status *in vivo* throughout the year is associated with changes in the human peripheral T cell compartment and may as such explain some of the seasonal variation in immune status which has been observed previously. Given that the current observations are limited to healthy adult males, larger population-based studies would be useful to validate these findings.

## Introduction

Vitamin D_3_ is traditionally associated with bone homeostasis and calcium metabolism. The extra-renal synthesis of 1,25-dihydroxyvitamin D_3_ [1,25(OH)_2_D_3_] by macrophages and other immune cells has re-invented the role of vitamin D_3_. In recent years, research efforts were also focused on understanding the immunemodulatory properties of vitamin D_3_. 1,25-dihydroxyvitamin D_3_ has been shown to influence the growth and differentiation of both the innate and acquired immune cells, as well as their functions such as cytokine production [1]–[3]. As such, there has been much interest to identify its therapeutic potential in autoimmune or inflammatory diseases.

Sources of vitamin D_3_ include dietary uptake (primarily fatty fish and cod liver oil) as well as cutaneous biosynthesis from UVB exposure causing 7-dehydrocholestrol to form previtamin D_3_ in the skin. Vitamin D_3_ is subsequently hydroxylated into 25-hydroxyvitamin D_3_ [25(OH)D_3_] by 25-hydroxylase in the liver. 25-hydroxyvitamin D_3_ is further hydroxylated by 1α-hydroxylase in the kidney into the biologically active metabolite, 1,25(OH)_2_D_3_ [4]. The main source of vitamin D_3_ derives from UVB-induced vitamin D_3_ production, accounting for 80–90% of circulating vitamin D_3_ [5].

The seasonal variation in vitamin D_3_ status in temperate and cold climates with reduced sunlight exposure during certain periods of the year is thought to be responsible for the high prevalence of vitamin D_3_ insufficiency among populations residing at higher latitudes [6]. Low wintertime vitamin D_3_ levels have been found partly accountable for the seasonal peak in influenza and URTI occurrence [7]–[9]. Moreover, reduced sun exposure and vitamin D_3_ status have been identified as risk factors for the development of autoimmune diseases. Epidemiological studies have implicated seasonality of birth as well as geographical variation in UV radiation and serum vitamin D_3_ levels as contributing factors to the prevalence of multiple sclerosis and insulin-dependent diabetes mellitus [10]–[15].

T cells are known targets for 1,25(OH)_2_D_3_ since they express vitamin D receptor [16], [17]. Upon T cell activation, the expression of vitamin D receptor is up-regulated, suggesting an important functional role for vitamin D_3_ in adaptive immunity. Both human *in vitro* and animal models revealed that vitamin D_3_ can suppress pro-inflammatory T helper (Th)1 and Th17 cytokine responses [18], [19], while enhancing the production of interleukin (IL)-4, IL-5 and IL-10, thereby promoting a Th2 and regulatory T cell (Treg) phenotype [20], [21]. Indeed, accumulating evidence supports the notion that vitamin D_3_ could favorably influence the course of certain autoimmune pathology by increasing the number of Treg [13], [15]. In addition, chemokine receptors expression is a determining factor in migration and localization of T lymphocytes during physiological and inflammatory responses [22], [23]. 1,25(OH)_2_D_3_ has been demonstrated to affect the homing capacity of the peripheral CD4^+^ T cell population *in vitro* and in an animal model [24], [25].

Taken together, the involvement of 1,25(OH)_2_D_3_ in the dynamics of T cell compartment warrants further investigation. Previously, we have found a down-regulation of Toll-like receptor (TLR)4-mediated proinflammatory cytokines production in association with an elevated vitamin D_3_ status in summer [26]. However, our current knowledge on the immunomodulatory role of vitamin D_3_ conveys limited information on how the adaptive immune response of healthy individuals varies in response to physiological changes in vitamin D_3_ status *in vivo* during the different seasons of the year. Intrigued by the strong epidemiological association between vitamin D_3_ deficiency and autoimmunity, and the proposed effects of 1,25(OH)_2_D_3_ on Treg, we investigated whether there is a seasonal variation in the composition of the peripheral T cell pool and the circulating Treg. A potential modification in these parameters may provide a better understanding on how sun exposure and vitamin D_3_ can act as candidate risk-modifying factors in certain autoimmune disorders.

## Materials and Methods

### Study subjects

Fifteen healthy male volunteers (median 36 years old, range 28–60; mean BMI 22.8 kg/m^2^, range 20.5–26.2) were recruited and followed up for one year. Body mass index (BMI) has been shown to be inversely related to vitamin D_3_ levels [27]. We have eliminated this confounder from our study since none of the 15 volunteers was obese (BMI>30 kg/m^2^). Venous blood was drawn from the subjects every three months, at the end of four consecutive seasons in 2009; February in winter, May in spring, August in summer and November in autumn. On the rare occasions that a participant reported on being unwell, the experiment would be postponed until one week post-recovery.

### Ethics Statement

The study was approved by the Ethical Committee on Human Experimentation of the Radboud University Nijmegen. A written consent was obtained from all participants in the study.

### Flowcytometry

Cells were phenotypically analyzed by five-color flow cytometry (Coulter Cytomics FC 500, Beckman Coulter, Fullerton, USA) using Coulter Epics Expo 32 software. PBMC as well as whole blood (after red cell lysis) were used for flow cytometric analysis. Peripheral blood mononuclear cells (PBMC) were isolated by density centrifugation on Ficoll-Hypaque (Pharmacia Biotech, Uppsala, Sweden). Cells were washed with PBS with 0.2% bovine serum albumin (BSA) before being labeled with fluorochrome-conjugated antibodies (mAb). After incubation for 20 minutes at room temperature, in the dark, cells were washed twice to remove unbound antibodies and analyzed. For cell surface staining, the following mAb were used: CD127 PC5- or PC7-labeled (RDR5; eBioscience, Uithoorn, The Netherlands), CD25-PE (M-A251), CD25-APC (2A3) CD45RA-FITC (HI100), CCR4-PC7 (1G1), CCR6-PE (11A9), CLA-FITC (HECA-452) (all from BD Biosciences, Breda, The Netherlands), CD3-ECD (UCHT1), CD4 ECD- or PC7-labeled (SFCI12T4D11), CD4-PC5 (13B8.2), CD8-ECD (SFCI21Thy2D3), CD8-PC5 (B9.11), CD27-PC5 (1A4CD27), CD45RA-ECD (2H4LDH11LDB9) CD45RO-ECD (UCHL1) (all from Beckman Coulter, Mijdrecht The Netherlands), CCR7-FITC (150503), CCR9-PE (112509) (both from R&D Systems, Minneapolis, USA), CD27-FITC (M-T271), CD45-PE (T29/33), CD45RA-PE (4KB5) (both from Dako, Glostrup, Denmark) and CD31 Alexa Fluor® 488 (WM59) (BioLegend, San Diego, USA). Appropriate isotype control mAbs were used for gate settings. The live gate was set based on the forward angle light scatter (FSCs) and the side angle light scatter (SSCs), and Annexin-V/PI staining.

For intracellular staining of FoxP3 and Ki-67, cells were fixed and permeabilized using Fix and Perm reagent (eBioscience) according to the manufacturer's recommendations. The following mAb were used for staining: anti-FoxP3 FITC- or PE- labeled (FCH101; eBioscience), anti-Ki-67-FITC (B56, BD Biosciences).

Intracellular staining of cytokines was performed after 4 hours stimulation with PMA (12.5 ng/ml) and ionomycin (500 ng/ml) in the presence of Brefeldin A (5 µg/ml; Sigma, Zwijndrecht, The Netherlands). Cells were fixed and permeabilized using Fix and Perm reagent (eBioscience) according to the manufacturer's recommendations. The following mAb were used for staining: anti-IFNγ-PC7 (4S.B3), anti-IL-17-Alexa Fluor® 647 (eBIO64DEC17) (both from eBioscience), and anti-IL-2-PE (MQ1-17H12) (BD Bioscience).

### Vitamin D_3_ measurement

Serum 25(OH)D_3_ was determined using high-performance liquid chromatography (HPLC) with ultraviolet (UV) detection, after prior extraction on small SepPak columns as previously described [28]. Tritiated 25(OH)D_3_, collected from the HPLC system during passage of the UV peak, was used to correct for procedural losses. Serum 1,25(OH)_2_D_3_ was measured using a radioreceptor assay (RRA) with prior extraction and chromatographic pre-purification with correction for recovery as previously described [29]. For 25(OH)D_3_, the within run precision was 2.6% at 69 nmol/l and between run precision was 6.2% at 69 nmol/l. For 1,25(OH)_2_D_3_, the within run precision was 10.6% at 115 pmol/l and between run precision was 17.2% at 69 nmol/l.

### Statistical analysis

Results were pooled and analyzed using SPSS 16.0 statistical software. Data given as means+SEM and the Analysis of Variance (ANOVA) was performed to assess overall variation. Where the ANOVA indicated a significant difference (p<0.05), the Friedman test using Graphpad PRISM software (Graphpad Prism Inc., version 4, CA, USA) was used to compare differences between groups (unless otherwise stated). The level of significance was set at p<0.05.

## Results

### Serum 25(OH)D_3_ and 1,25(OH)_2_D_3_ levels are increased during summer

**Figure 1:**
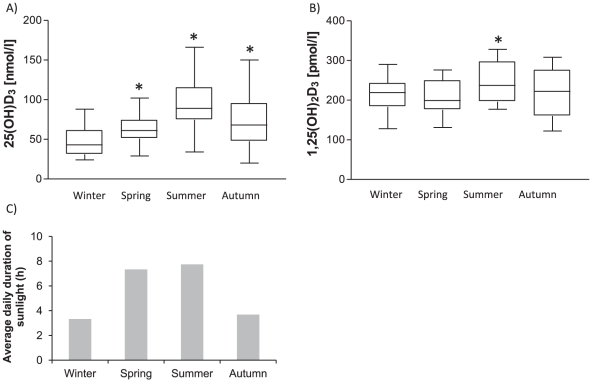
Seasonal variation in serum vitamin D_3_ levels and the amount of daylight. Median serum A) 25(OH)D_3_ and B) 1,25(OH)_2_D_3_ concentrations of 15 healthy volunteers during each of the four seasons. C) Duration of daylight in the study region in a month prior to serum vitamin D_3_ concentration assay (source: the Royal Netherlands Meteorological Institute). * p<0.05 as compared to winter.

First, we determined serum concentrations of both 25(OH)D_3_ and 1,25(OH)_2_D_3_ in 15 healthy volunteers (median 36 years old, range 28–60) through winter (December to February), spring (March to May), summer (June to August) and autumn (September to November). The median concentration of 25(OH)D_3_ varied between the four seasons and was doubled from 43 nmol/l in winter to 89 nmol/l in summer (Figure 1A). Also, the median serum concentration of 1,25(OH)_2_D_3_ raised significantly from 219 pmol/l in winter to 237 pmol/l in summer (Figure 1B). These observed trends paralleled the amount of sunlight in the study region. Likewise, there is considerable seasonal difference in ultraviolet B (UVB) radiation in the study region [30]. The total duration of daylight in a month prior to vitamin D_3_ measurement were 103 hours and 240 hours in winter and summer respectively, which worked out to an average daily duration of 3.3 hours in winter and 7.7 hours in summer (Figure 1C).

### Seasonal variation in peripheral blood T cell subset numbers associated with vitamin D3 levels

**Figure 2:**
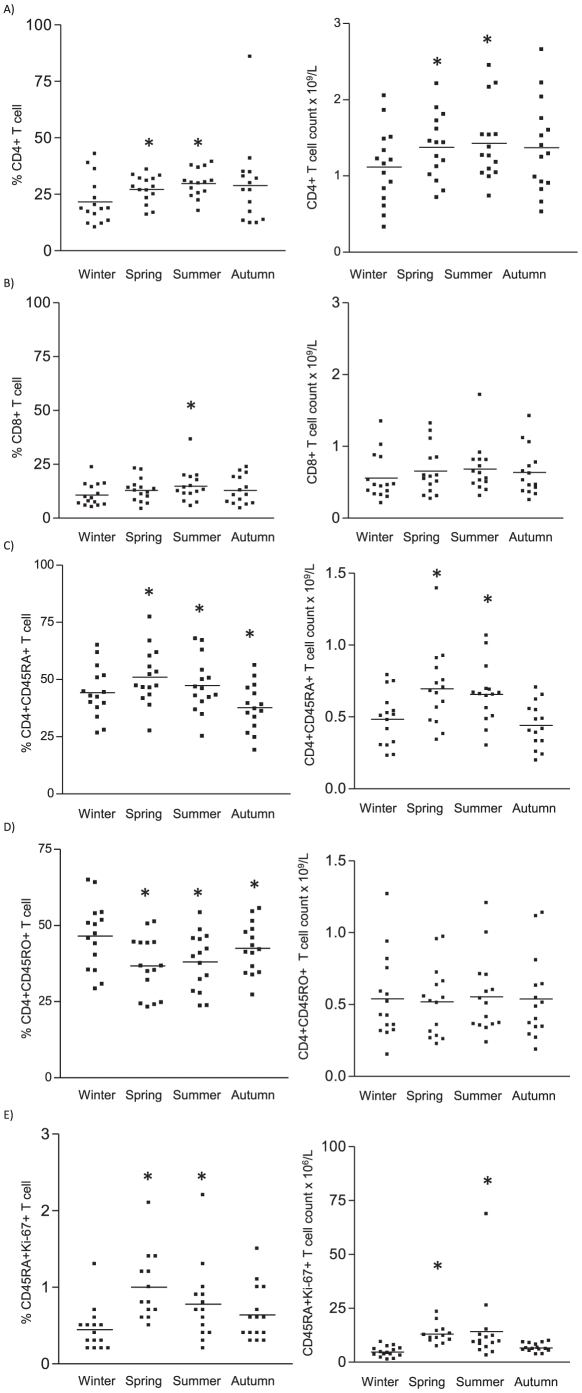
Peripheral T cell (subset) numbers throughout the four seasons. A) Percentage (of live gate) and absolute numbers of CD4^+^ T cells. B) Percentage (of live gate) and absolute numbers of CD8^+^ T cells, over time. C) Percentage (within CD4^+^ T cells) and absolute counts, of CD4^+^CD45RA^+^ T cells. D) Percentage (within CD4^+^ T cells) and absolute counts, of CD4^+^CD45RO^+^ T cells. E) Percentage and absolute counts of Ki-67-expressing CD4^+^CD45RA^+^ T cells. Whole blood samples obtained from 15 healthy volunteers during each season were analyzed for the respective markers using flow cytometry. Ki-67 analysis was performed on PBMC. Data show results of viable cells from 15 healthy donors. * p<0.05 as compared to winter.

Next, we investigated whether seasonal variation in vitamin D_3_ status was associated with changes in the peripheral T cell pool, by performing flowcytometric analysis on blood samples obtained during the different seasons of the year (Figure S1). In spring and summer months when serum vitamin D_3_ levels were elevated, the percentage as well as the absolute CD4^+^ T cell counts were significantly raised as compared to winter (Figure 2A). For CD8^+^ T cells, this effect was less outspoken (Figure 2B).

The composition and size of the naïve and memory T cell pools are regulated by cytokines and T cell receptor (TCR) signalling from contact with major histocompatibility complex (MHC). Naïve T cells predominately express CD45RA and memory T cell express CD45RO. Interestingly, during spring and summer, we observed a relative increase in the percentage of CD4^+^CD45RA^+^ T cells (Figure 2C), with a corresponding drop in CD4^+^CD45RO^+^ T cell percentage (Figure 2D). Also, absolute CD4^+^CD45RA^+^ T cell counts were increased in spring and summer months, while the number of CD4^+^CD45RO^+^ T cells was not significantly changed. To investigate whether the increase in peripheral CD4^+^CD45RA^+^ T cells as observed in spring and summer could be attributed to recent thymic emigration or a higher proliferative capacity; we stained cells with Ki-67 and CD31. Ki-67 is a nuclear protein associated with cellular proliferation, while CD31 has been used as a marker for recent thymic emigrants [31]. In spring and summer, an increased Ki-67-expressing population was found within the CD4^+^CD45RA^+^ T cells (Figure 2E). On the other hand, there were no significant differences in both the frequency of CD4^+^CD45RA^+^ T cell expressing CD31 as well as their level of expression between winter and summer (data not shown).

**Figure 3:**
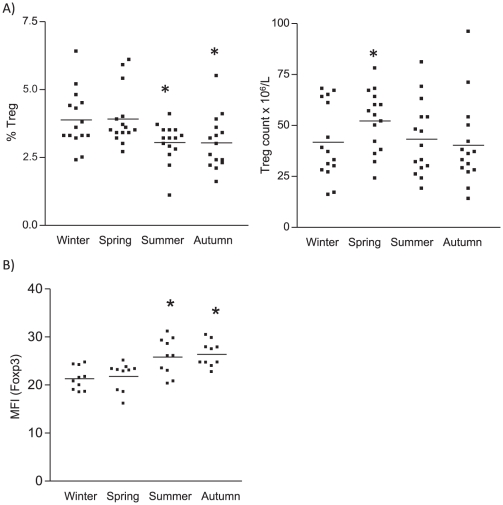
Seasonal variation in numbers and Foxp3 expression of Treg during the four seasons. A) Percentage (within CD4^+^ T cells) and absolute numbers of CD4^+^CD25^hi^CD127^−^ Treg and B) level of Foxp3 expression (mean fluorescence intensity; MFI). Whole blood and PBMC isolated from 15 healthy volunteers during each season were analyzed for the respective markers using flowcytometry. Data show results from 15 healthy donors. * p<0.05 as compared to winter.

The increase in vitamin D_3_ levels found in summer, as compared to winter was paralleled by a reduction in the percentage of CD25^hi^CD127^−^ Treg within the CD4^+^ T cell population (Figure 3A), however the absolute Treg numbers were not associated with the variation in vitamin D_3_ levels. Of note, the level of expression (mean fluorescence intensity, MFI) of Foxp3 by the peripheral regulatory T cell population was increased in summer (Figure 3B).

### Seasonal variation in homing potential of peripheral blood CD4^+^ T cells

Peripheral T cell trafficking is regulated by specific chemokine receptors which are selectively expressed by the various T cells subsets. As 1,25(OH)_2_D_3_ has been demonstrated to affect the homing capacity of the peripheral CD4^+^ T cell population *in vitro* and *in vivo*, we wondered if we could detect seasonal variation in homing receptors expression. We looked at the expression of homing markers on CD4^+^ T cells, as well as more specifically on the Treg population, and included chemokine receptors associated with migration to the skin (CCR4, CCR6 and CLA), gut (CCR9) and lymphoid tissues (CCR7).

**Figure 4:**
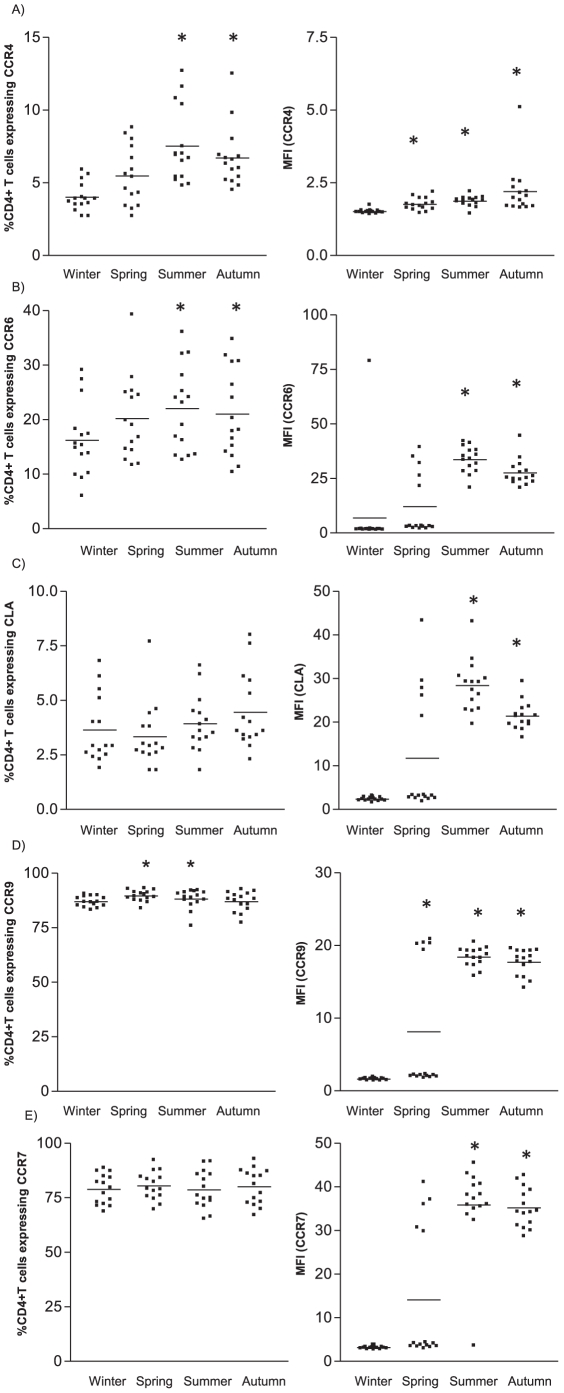
Skin, lymphoid tissue and gut homing receptor expression on CD4^+^ T cells. Percentage and level of expression (MFI) of A) CCR4, B) CCR6, C) CLA, D) CCR7 and E) CCR9 by CD4^+^ T cells during the different seasons of the year. Whole blood from 15 healthy volunteers during each season was analyzed for the respective markers using flow cytometry. Data show results from 15 healthy donors. * p<0.05 as compared to winter.

In summer, an increased skin homing potential of CD4^+^ T cells was observed compared to winter, given that the percentage of CD4^+^ T cells expressing CCR4 and CCR6 (Figure 4A,B) was significantly increased together with elevated expression levels (MFI) of CCR4, CCR6 and CLA (Figure 4A–C). Also, the percentage of CD4^+^ T cells expressing the gut homing marker CCR9 was increased in summer, as well as the level of expression (Figure 4D). Similar observations were seen in the expression level of the chemokine receptor associated with lymphoid tissue homing, CCR7 (Figure 4E).

**Figure 5:**
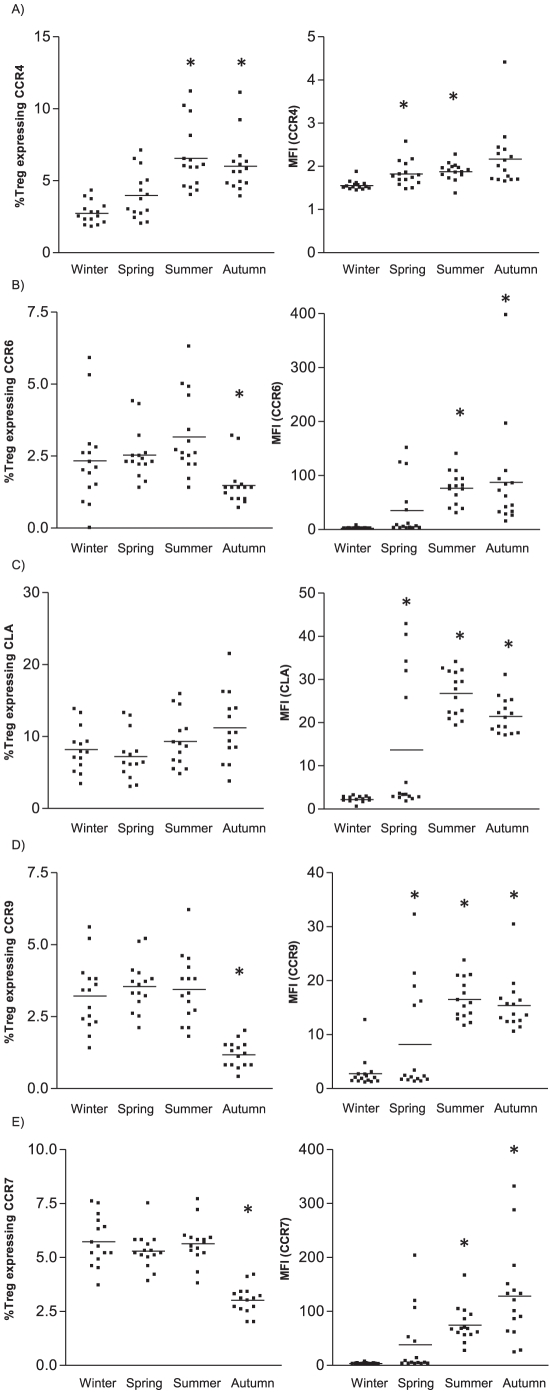
Skin, lymphoid tissue and gut homing receptor expression on CD4^+^CD25^hi^CD127^−^ regulatory T cells. Percentage of Treg (within CD4^+^ T cells) and their level of expression (MFI) of A) CCR4, B) CCR6, C) CLA, D) CCR7 and E) CCR9 during the four seasons of the year. Whole blood from 15 healthy volunteers during each season was analyzed for the respective markers using flow cytometry. Data show results from 15 healthy donors. * p<0.05 as compared to winter.

The skin homing potential of the regulatory T cell subset mirrored that of the whole peripheral CD4^+^ T cell population. Notably, in especially in summer Treg displayed a heightened skin homing potential as seen by a significantly increased frequency of CCR4-expressing Treg (Figure 5A), and higher expression levels of CCR4, CCR6 and CLA (Figure 5A–C), when compared to winter. The level of expression (MFI) of chemokine receptors involved in gut homing, CCR9 (Figure 5D) and lymphoid tissue homing, CCR7 (Figure 5E) were also increased in summer.

### Reduced proinflammatory cytokine production by peripheral blood T cells in summer

**Figure 6:**
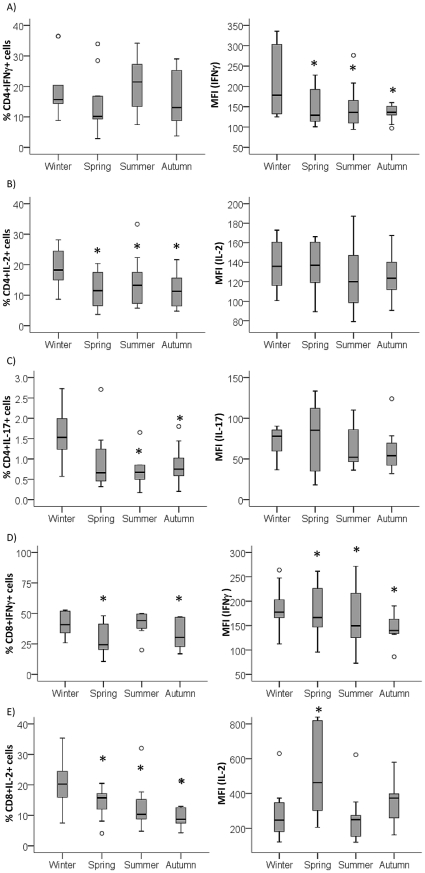
Seasonal variation in cytokine profile of CD4^+^ and CD8^+^ T cells. Percentage and the level of production on a per cell basis (MFI) of A) IFNγ, B) IL-2 and C) IL-17 by CD4^+^ T cells; and of D) IFNγ and E) IL-2 by CD8^+^ T cells analyzed by flow cytometry. PBMC isolated from 10 healthy volunteers and intracellular staining for cytokines was performed after the cells were stimulated with PMA plus ionomycin in the presence of brefeldin A. CD4^+^ T cells were defined as CD3^+^CD8^−^. Data show results from 10 healthy donors. * p<0.05 as compared to winter.

Intrigued by the increased CD4^+^ T cell numbers in spring and summer, we also looked at functional characteristics of the cells by examining the cytokine-producing capacity of CD4^+^ T cells using intracellular cytokine staining for interferon (IFN)γ, IL-2 and IL-17. There was no significant effect on the percentage of IFNy-producing CD4^+^ T cells (Figure 6A), but the level of expression was lowered in summer (p<0.05). The percentages of IL-2 and IL-17- secreting CD4^+^ T cells were reduced in summer (Figure 6B and 6C), with unchanged levels of production on a per cell basis.

Also for CD8^+^ T cells we found lowered levels of IFNy production from spring to autumn (Figure 6D). The percentage of CD8^+^ T cells producing IL-2 was significantly reduced from spring to autumn (Figure 6E); expression levels were increased during spring.

## Discussion

There is growing evidence that vitamin D_3_ plays a pivotal role in infections and autoimmune diseases. Whilst UV-induced vitamin D_3_ production serves as the main source of vitamin D_3_ in the body [5], it is not apparent whether seasonal variation in vitamin D_3_ can impact T cell immunity. We show for the first time that physiological elevation in vitamin D_3_ concentrations during summer is paralleled by changes in the peripheral T cell composition, with a notable shift in the naïve and memory CD4^+^ T cell balance as a consequence of increased proliferation of naïve CD4^+^CD45RA^+^ T cells.

By virtue of its stability and long half-life, 25(OH)D_3_ is the vitamin D metabolite that best reflects the vitamin D_3_ status [32]. Here, we found a significant difference between winter (December to February) and summer (June to August) 25(OH)D_3_ levels. Serum 1,25(OH)_2_D_3_ concentrations were also higher in summer as compared to winter. In our cohort of 15 subjects residing at 52°N from the Equator, this variation correlated with the amount of sunlight and ultraviolet B radiation received in the study region. Vitamin D_3_ insufficiency at high latitudes has been implicated in the prevalence of autoimmune diseases such as multiple sclerosis and insulin-dependent diabetes [33], [34]. Therefore, we investigated whether the peripheral T cell compartment might vary with physiological changes in vitamin D_3_ status throughout the year.

We found higher percentages of peripheral CD4^+^ and CD8^+^ T cells concomitant with a heightened vitamin D_3_ status during summer. Of note, we observed a higher proportion of CD4^+^CD45RA^+^ naïve T cells in the spring/summer months with a corresponding drop in the percentage, but not in the absolute number, of CD4^+^CD45RO^+^ memory T cells. When investigated further, the expansion of CD4^+^CD45RA^+^ naïve T cells resulted from an increased proliferative capacity as seen by a higher absolute cell count and an increased population expressing the proliferative marker, Ki-67. One of the key targets of 1,25(OH)_2_D_3_ are the CD4^+^ T cells. *In vitro*, 1,25(OH)_2_D_3_ inhibits T cell proliferation [35], [36]. Though few studies examined the differential effects on naïve and memory T cells, the inhibitory effect has been found to be more pronounced in the memory T cell compartment [37].

1,25(OH)_2_D_3_ exerts a marked inhibitory effect on cells of the adaptive immune system and it has been consistently described that 1,25(OH)_2_D_3_ inhibits cytokines such as IFNγ [21], [38] and IL-17, as well as IL-2 [19], [39], both under *in vitro* conditions and in animal models. Our data reveal that in healthy adult males residing at 52°N from the Equator, the percentages of IL-17- and IL-2-producing CD4^+^ T cells were down-regulated in summer and the IFNy expression levels in both CD4^+^ and CD8^+^ T cells were also reduced.

Regulatory T cells are characterized by a constitutively high expression of the transcription factor, Foxp3. We observed that, although the percentage of peripheral Treg was lower in summer as compared to winter, there was no correlation between absolute numbers of Treg and vitamin D_3_ levels. This is in concert with findings of Smolders et al, who failed to detect a correlation between Treg numbers and serum 25(OH)D_3_ levels in patients with multiple sclerosis [40]. Of note, they did find that higher 25(OH)D_3_ levels were associated with improved suppressive function. This fits our data on increased expression of Foxp3 in the Treg during summer. Morales-Tirado et al reported that *in vitro*, 1,25(OH)_2_D_3_ enhanced Treg function by increasing the expression of Foxp3 and that this was shown to be associated with modulation of cell cycle progression by vitamin D_3_ [41].

T cell migration is determined by the presence of specific selectins, chemokine receptors and integrins. Homing receptors are selectively expressed and regulated in different T cell subsets [23], [42]. Our results are suggestive of a vitamin-D_3_ associated up-regulation of skin, gut- and lymphoid tissue- homing expression on CD4^+^ T cells, including Treg. Although not previously described in the context of physiological variation, 1,25(OH)_2_D_3_ has been reported to influence certain skin homing markers in human T cells. *In vitro*, it has been shown that addition of 1,25(OH)_2_D_3_ resulted in induction of CCR10, inhibition of CLA, but not CCR4 and CCR6 expression [38], [43]. In our study, we found that during summer an increased frequency of CCR4-expressing cells as well as an increased level of expression (mean fluorescence intensity; MFI) of CCR4, CCR6 and CLA. These data suggest that in summer CD4^+^ T cells, including Treg, are better equipped to migrate to the skin. Also, we observed higher levels of CCR9 and thus heightened potential to migrate to the gut. Previously, 1,25(OH)_2_D_3_ was described not to affect gut-homing markers [25]. However, it should be appreciated that the physiological up-regulation of vitamin D_3_ levels by UV light through the skin is likely to yield distinct effects from those obtained through supraphysiological doses employed in these *in vitro* studies.

In the present study, we assessed a homogenous study population (healthy, adult males of normal BMI) to establish if and how the human peripheral T cell compartment varies with the season. Unique to previous *in vitro* and *in vivo* studies examining the role of 1,25(OH)_2_D_3_ on T cells, our current results suggest that physiological variation in serum vitamin D_3_ levels throughout the four seasons might influence CD4^+^ and CD8^+^ T cell homeostasis and homing behavior. Given that serum 25(OH)D_3_ levels can be affected by various factors, our observations warrant future validation in a larger and more diverse population cohort to identify any possible differences in adaptive immune responses among the extreme of ages and different genders. Nevertheless, our data provide insight on previous epidemiological findings regarding the prevalence of certain autoimmune diseases and infections, which have been attributed to seasonal variation in sun exposure and serum 25(OH)D_3_ levels [10]–[12],[44]. Although not as extensively reported as vitamin D_3_ status, certain hormones and corticosteroids such as catecholamine and aldosterone seem to vary with seasons as well [45], [46]. It would be of interest to find out if these factors are associated with changes in immunological characteristics of T cell.

In conclusion, we have demonstrated for the first time the existence of variations in adaptive immunity throughout the four seasons of the year in association with physiological changes in serum 25(OH)D_3_ levels *in vivo*. These novel findings further our understanding on the seasonal variability between vitamin D_3_ and human peripheral T cell composition, and support the basis for conducting larger population-based studies to investigate the benefits of vitamin D_3_ supplementation in temperate regions during winter.

## Supporting Information

Figure S1 **Gate setting for CD4^+^ and CD8^+^ T cells gated on CD45^+^ cells; and CD4^+^CD45RA^+^ T cells, CD4^+^CD45RO^+^ T cells and CD4^+^CD25^hi^CD127^−^ regulatory T cells gated on CD4^+^ T cells.** Dotplots show surface staining for markers performed on whole blood.
(EPS)
